# An Integrative Review on the Main Flavonoids Found in Some Species of the Myrtaceae Family: Phytochemical Characterization, Health Benefits and Development of Products

**DOI:** 10.3390/plants11202796

**Published:** 2022-10-21

**Authors:** Vinícius Tadeu da Veiga Correia, Pâmela Rocha da Silva, Carla Mariele Silva Ribeiro, Ana Luiza Coeli Cruz Ramos, Ana Carolina do Carmo Mazzinghy, Viviane Dias Medeiros Silva, Afonso Henrique Oliveira Júnior, Bruna Vieira Nunes, Ana Luiza Santos Vieira, Lucas Victor Ribeiro, Ana Cardoso Clemente Filha Ferreira de Paula, Júlio Onésio Ferreira Melo, Camila Argenta Fante

**Affiliations:** 1 Departamento de Alimentos, Faculdade de Farmácia, Campus Belo Horizonte, Universidade Federal de Minas Gerais, Belo Horizonte 31270-901, MG, Brazil; 2 Departamento de Ciências Exatas e Biológicas, Campus Sete Lagoas, Universidade Federal de São João del-Rei, Sete Lagoas 36307-352, MG, Brazil; 3 Departamento de Ciências Agrárias, Campus Bambuí, Instituto Federal de Educação, Ciência e Tecnologia de Minas Gerais, Bambuí 38900-000, MG, Brazil

**Keywords:** bioactive compounds, *Psidium cattleianum*, *Myrciaria floribunda*, *Campomanesia xanthocarpa*, *Plinia cauliflora*, *Syzygium cumini*

## Abstract

This integrative review aims to identify the main flavonoids present in some species of the Myrtaceae family. Studies published between 2016 and 2022 were selected, specifically those which were fully available and written in Portuguese, English, or Spanish, and which were related to the fruits araçá (*Psidium cattleianum*), cambuí (*Myrciaria floribunda*), gabiroba (*Campomanesia xanthocarpa*), jabuticaba (*Plinia cauliflora*), and jambolan (*Syzygium cumini*). Scientific studies were gathered and selected in Google Scholar, Scielo, and Science Direct indexed databases, out of which 14 were about araçá, 7 concerned cambuí, 4 were about gabiroba, 29 were related to jabuticaba, and 33 concerned jambolan, when we observed the pre-established inclusion criteria. Results showed that the anthocyanins, such as cyanidin, petunidin, malvidin, and delphinidin, were the mostly identified class of flavonoids in plants of the Myrtaceae family, mainly relating to the purple/reddish color of the evaluated fruits. Other compounds, such as catechin, epicatechin, quercetin, and rutin were also identified in different constituent fractions, such as leaves, peel, pulp, seeds, and in developed products, such as jams, desserts, wines, teas, and other beverages. It is also worth noting the positive health effects verified in these studies, such as anti-inflammatory qualities for jambolan, antidiabetic qualities for gabiroba, antioxidant qualities for araçá, and cardioprotective actions for jabuticaba, which are related to the presence of these phytochemicals. Therefore, it is possible to point out that flavonoids are important compounds in the chemical constitution of the studied plants of the Myrtaceae family, with promising potential in the development of new products by the food, chemical, and pharmaceutical industries due to their bioactive properties.

## 1. Introduction

Myrtaceae is a family comprising 121 genera and 5800 plant species, occurring mainly in tropical and subtropical regions of the world, being a predominant group in the Brazilian Atlantic Forest [1]. Several species have significant economic and technological value, such as eucalyptus (*Eucalyptus* spp.), used in the production of wood and flavoring agents [2], as well as numerous fruit plants, such as the araçazeiro (*Psidium cattleianum* Sabine) [3], cambuizeiro (*Myrciaria floribunda* (H. West ex Willd.) O. Berg) [4], gabirobeira (*Campomanesia xanthocarpa* (Mart.) O. Berg) [5], jambolão (*Syzygium cumini* (L.) Skeels) [6], and jabuticabeira (*Plinia cauliflora* (Mart.) Kausel) [7], studied mainly for their nutritional, sensory, and bioactive properties.

Various species of the Myrtaceae family, when processed, provide important products, such as essential oils, dyes, and food products, and can be also employed in traditional medicine [8,9,10,11]. This medicinal potential has been experimentally proven and associated with anti-rheumatic, antidiabetic, antimicrobial, diuretic, and digestive system regulatory activities, among others health beneficial activities, and has been evaluated in different experimental models both in vitro and in vivo [5,6,7,12].

The range of utilities provided by these plants may be related to the presence of phytochemicals, such as flavonoids, which are the main compounds of interest in this study. These compounds help in the prevention of various chronic non-communicable diseases, such as cardiovascular pathologies, oxidative stress, certain types of cancers, atherosclerosis, diabetes, Alzheimer’s disease, cataracts, and other respiratory disorders, and are concentrated in different parts of the plant [13]. Flavonoids are phenolic compounds of plant origin and have several biological properties with antioxidant, anti-inflammatory, antibacterial, antiallergic, and vasodilatory action [13].

In this context, fruit trees of the genera *Psidium, Myrciaria, Campomanesia, Plinia*, and *Syzygium* stand out for their phytochemical composition with a multitude of bioactive compounds, characterized by the production of flavonoids, water-soluble and condensed tannins, saponins, mono- and sesquiterpenes, and triterpenoids, among others [14].

The genus *Psidium* originates from tropical and subtropical America, which has great biological and economic importance in Brazil. This group includes several species of trees and shrubs, with emphasis on the guava tree (*Psidium guajava* L.) and araçá tree (*P. cattleianum*), plants geographically distributed in several regions of Brazil [15].

**Figure 1:**
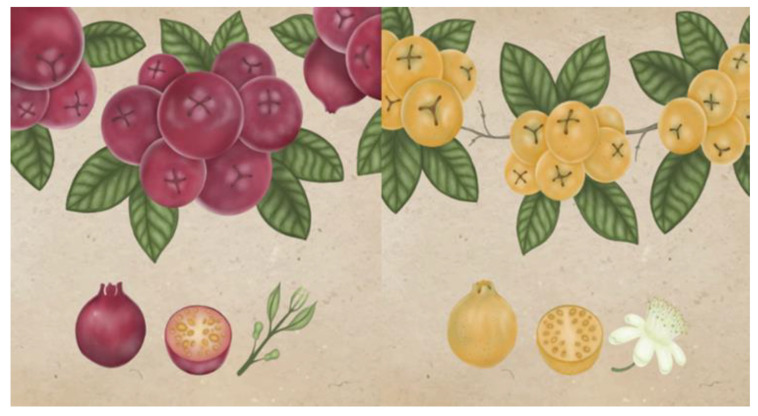
Red and yellow araçá (*P. cattleianum*). Illustration made by the Ribeiro, L.V. (2022).

Popularly, the fruits of the araçá tree are known as araçá, yellow araçá, red araçá, araçá-de-comer, araçá-da-praia, araçá-de-coroa, and araçá-do-mato. Despite the distinction of names, varieties and color, the fruits are characterized by having an ovoid shape, translucent pulp, and a kernel full of seeds, which can vary from approximately 22 to 250 units (Figure 1) [16,17,18].

Araçá has high agro-food potential due to its chemical composition. Among the compounds found in this fruit, organic acids, carotenoids, and flavonoids stand out [19]. In terms of the proximate composition of the fresh fruit, the nutrient contents are found in the following concentrations: 81.73–84.9 g of moisture; 4.32–10.01 g of carbohydrates; 3.87–6.14 g of fiber; 0.63–1.50 g of minerals; 0.75–1.03 g of proteins, and 26.8 kcal of energy [18].

Food technology allows the incorporation of araçá into a multitude of products in order to make it available on the market, and it is found in the form of sweets, jams, and flour, benefiting producers and adding economic value to certain communities [20]. In scientific study, araçá was used in the development of jams [20], yoghurts [21], chewable bullets [22], and bulk candy [10].

**Figure 2:**
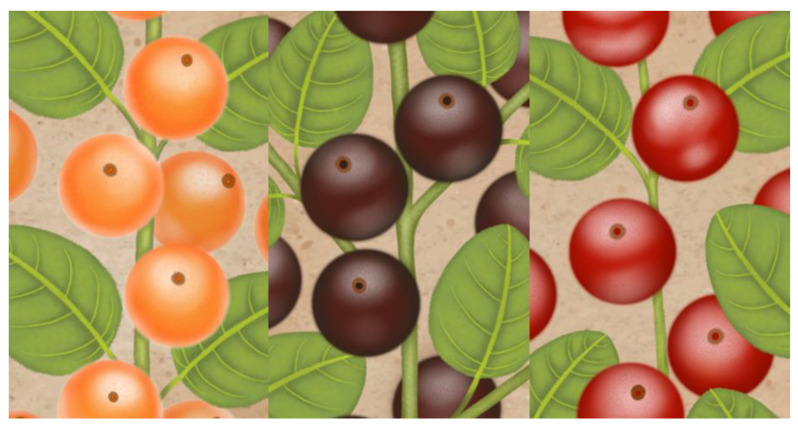
Orange, purple, and red cambuí fruits (*M. floribunda*). Illustration made by the Ribeiro, L.V. (2022).

Part of the genus *Myrciaria*, the cambuí tree is a medium-sized tree (with a height of 6–14 m) and a rounded crown, distributed throughout the northeast and north of South and Central America. Its fruits are characterized by being shiny, fleshy, and juicy elliptical berries, which can have a diverse color, ranging from orange to dark purple (Figure 2), a characteristic dependent on the stage of maturation and the variety evaluated [23].

In terms of physical properties, the cambuí fruit can reach up to 13 mm in diameter, with an average weight of 0.86 g and a number of seeds ranging from 1–3 units. When ripe, they can be consumed fresh or industrialized, in the form of sweets, jams, juices, and other dry or freeze-dried products. The contents of its physicochemical composition are equal to 3.53 for pH, 13.42 °Brix for the content of soluble solids, 4.03% of citric acid for titratable acidity, and 3.49 for the ratio between soluble solids and titratable acidity [4].

Cambuí presents a succulent pulp, a sweet and astringent flavor, and is characterized by having high concentrations of sugars and excellent levels of vitamin C (129.43 mg of ascorbic acid/100 g^−1^). Additionally, they are fruits that have antioxidant compounds in their constitution, especially carotenoids and flavonoids [24,25].

The gabiroba tree, of the genus *Campomanesia*, is a fruit tree native to Brazil, distributed in the territory of the south, center-west, and northeast regions, presenting an erect habit and reaching between 4 and 25 m in height. Its leaves are used in traditional medicine and in the preparation of teas, as well as being employed in the treatment of inflammation, kidney diseases, and hypercholesterolemia [26].

**Figure 3:**
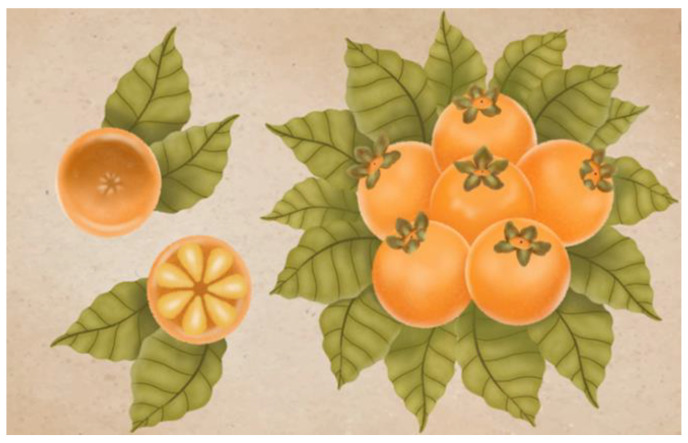
Gabiroba (*C. xanthocarpa*). Illustration made by the Ribeiro, L.V. (2022).

Gabiroba tree fruits are popularly known as gabiroba and have sensory characteristics, such as a sweet acid flavor, juicy pulp, yellow-orange color, and thin skin (Figure 3). Among the various compounds already identified in gabiroba, the classes of flavonoids, carotenoids, and vitamins stand out, which have a high antioxidant and anti-inflammatory capacity [27].

Due to its sensory and bioactive properties, gabiroba has already been used as a raw material in the development of jams [28], added in a dehydrated form into chocolate bars [29], and its seeds have been evaluated for their antidiabetic and hypolipidemic potential [5], despite the industrial exploitation of gabiroba still being in its initial stages.

Jambolan, of the genus *Syzygium*, is popularly known as jamelão, cereja, jalão, kambol, jamun, azeitona-do-nordeste, ameixa-roxa, murta, guapê, jambuí, azeitona-da-terra, baga-de-freira, brinco-de-viúva, or jambalau [30]. It is a plant native to India, but one which is currently widespread in different Brazilian regions; it reaches 10 m in height and has a leafy crown [31].

**Figure 4:**
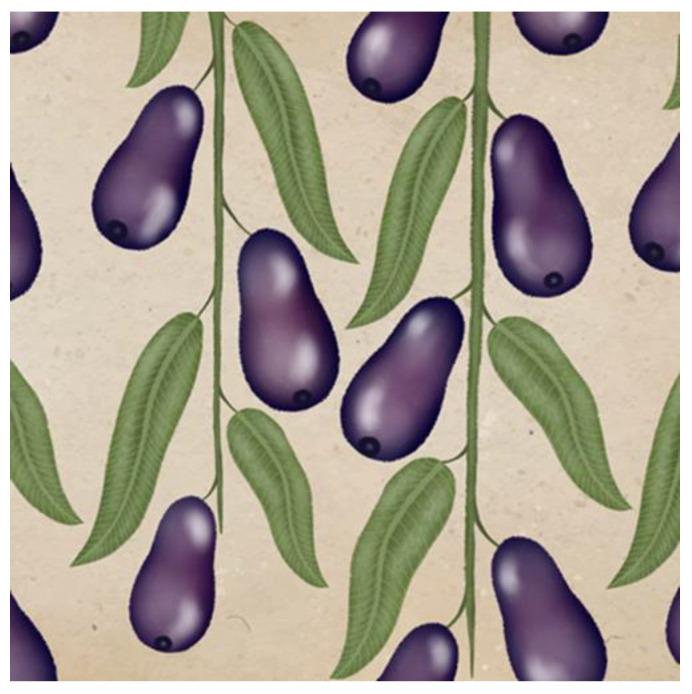
Jambolan (*S. cumini*). Illustration made by the Ribeiro, L.V. (2022).

Fruits are small and ovoid in shape. Before ripening, they are characterized by their green color and, when ripe, they may have a purplish black hue [32] (Figure 4). Due to its varied chemical composition, jambolan represents a potential raw material for the development of new fermented products, such as wines, liqueurs, and spirits, due to the significant levels of sugars. Additionally, it can also be used in the development of juices, jams, and yoghurts, as a way of preserving the fruits [33].

Studies involving jambolan are mainly related to its physicochemical and therapeutic properties, since they present expressive concentrations of flavonoids, carotenoids, resveratrol, and other polyphenols. Lago et al. [34] mention that the mineral, fiber, and lipid contents of these fruits are about 0.3%, proteins are equal to 0.7%, carbohydrates to are equal to 10.7% and 88% of the fruit is moisture.

**Figure 5:**
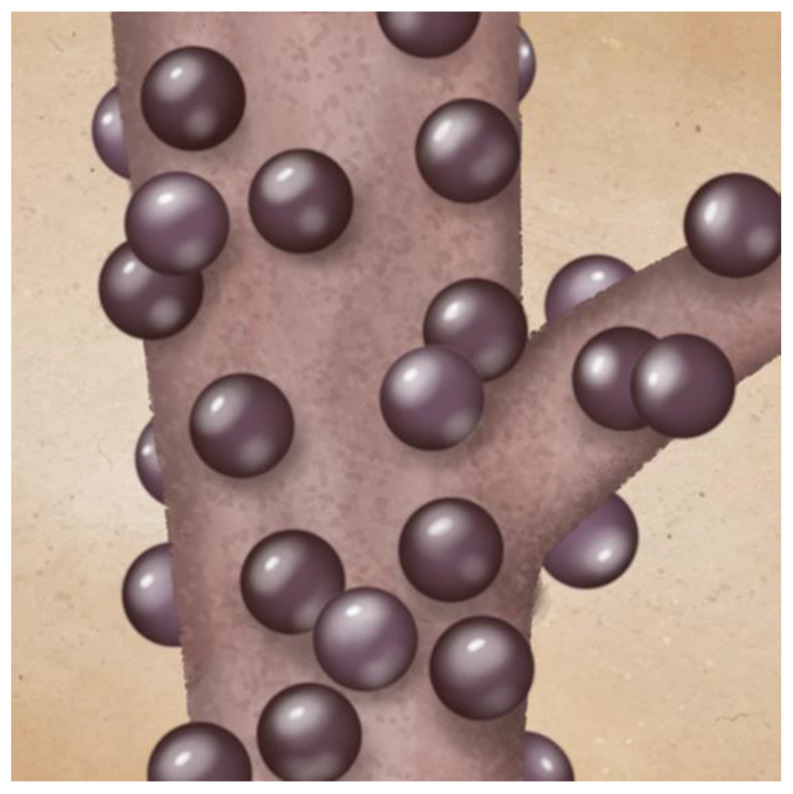
Jabuticaba (*P. cauliflora*). Illustration made by the Ribeiro, L.V. (2022).

Among the Brazilian wild fruit trees, the jabuticaba tree is of a great economic importance. It is a medium-sized plant (with an average of 6 to 9 m in height), with a varied, dense crown and a smooth reddish-yellow stem [35]. Its fruits have a globose berry shape, with diameters varying between 2 and 3.5 cm and red, purple, or black peels. Its pulp has a whitish color, a mucilaginous appearance, and a bittersweet flavor, with one to four seeds [36] (Figure 5).

Jabuticaba has high mineral concentrations, such as iron, copper, and manganese, as well as vitamin C; however, it stands out in the research scenario for its significant concentration of phenolic compounds, mainly anthocyanins, which are concentrated mainly in its peel, which is usually discarded and sees little use industrially [37].

According to Batista et al. [38], consumption of jabuticaba peel can contribute to the improvement of gastrointestinal tract functioning and can protect the liver against the action of certain free radicals, due to the abundance of fiber and antioxidant compounds, such as flavonoids. In this scenario, the exploration of the bioactive and technological potential of jabuticaba is extremely important and becomes a promising alternative product, aimed at the development of food and/or drugs and the reduction, at an environmental level, of the waste generated in the industrialization of these fruits.

Considering the large number of species of the Myrtaceae family and their importance, it is evident that there is still much to be studied, especially in relation to their chemical constituents. Therefore, the objective of this study was to carry out an integrative review identifying the main flavonoids present in *Psidium cattleianum, Myrciaria floribunda, Campomanesia xanthocarpa, Plinia cauliflora,* and *Syzygium cumini.*

## 2. Results and Discussion

**Figure 6:**
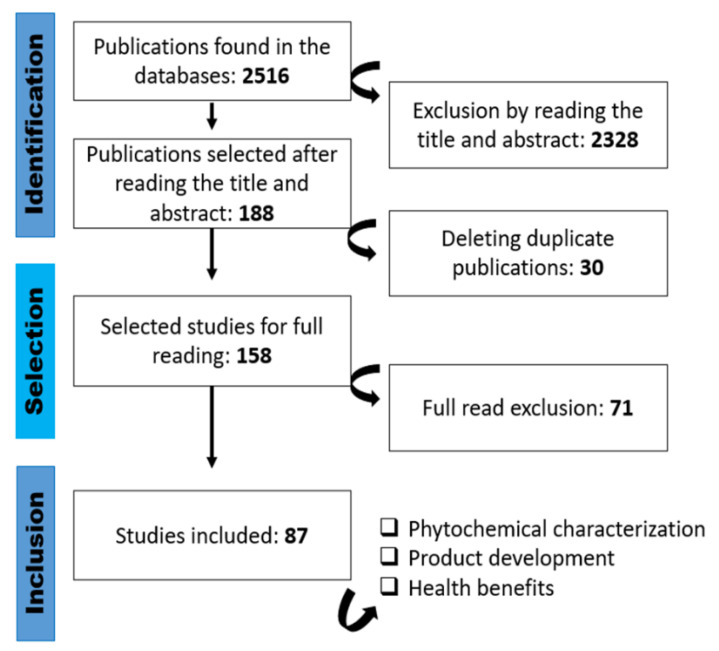
Flowchart of the selection of studies by stages (Authors, 2022).

Through the search strategies, 2516 scientific works were identified in the aforementioned databases, with the selection process shown in Figure 6.

Out of these studies, 2358 were excluded by the previous analysis of the title and abstract since they did not answer the guiding question or were found to be duplicated in different research bases. After a complete reading of 158 works, 87 articles published between 2016 and 2022 were selected to compose this integrative review, with an emphasis on the phytochemical characterization of vegetables, beneficial effects on human health, and product development with the maintenance of bioactive compounds.

**Figure 7:**
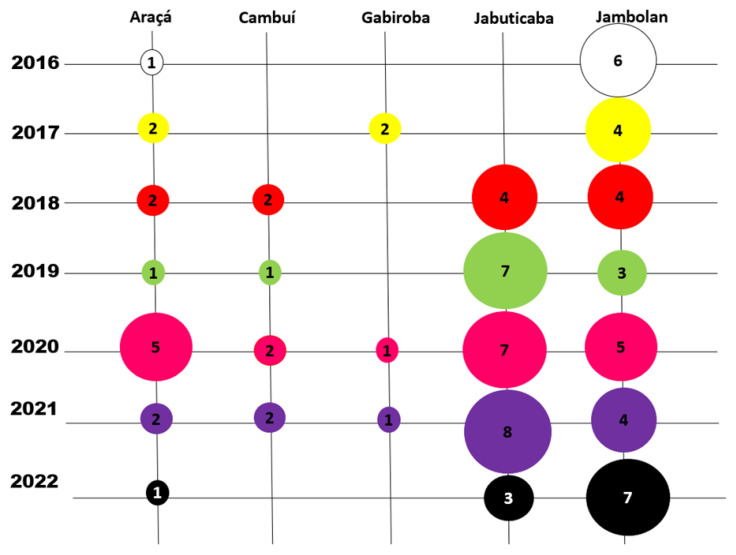
Bubble chart showing the number of selected publications in a specific yearly period (Authors, 2022).

As shown in Figure 7, 14 manuscripts comprised research on araçá, 7 on cambuí, 4 on gabiroba, and 29 on jabuticaba, while the remainder related to jambolan, with 33 articles selected following the pre-established inclusion criteria. Regarding the period of publications, the scores (percentage and sample number) are as follows: articles published in 2022 (12.64%, *n* = 11), in 2021 (19.55%, *n* = 17), in 2020 (22.98%, *n* = 20), in 2019 (13.79%, *n* = 12), in 2018 (13.79%, *n* = 12), in 2017 (9.20%, *n* = 8), and in 2016 (8.05%, *n* = 7).

### 2.1. Phytochemical Characterization

Bioactive compounds or phytochemicals are substances derived from the secondary metabolism of plants, with beneficial properties for human health [39]. Flavonoids stand out among these compounds as an extensive class of antioxidants found in different parts of the plant, such as the fruits and their different constituent fractions (peel, pulp, and seed), leaves, branches, and roots [40].

Table 1 summarizes the data referring to the selected articles that had the theme of phytochemical characterization of plants of the Myrtaceae family. It is observed that most studies comprised of assays with jambolan, jabuticaba, and araçá, using different techniques and methodologies to identify and quantify flavonoids.

For the identification and/or quantification of these bioactive constituents in the different extracts evaluated, the following methods were employed: spectrophotometry [3], LC-DAD-ESI-MS/MS [47], UPLC/QTOF/MS [42,50], PS-MS [4], liquid chromatography [56], and HPLC-DAD-ESI/MS [37,58,59], among others.

Catechin and quercetin were the prominent flavonoids in the evaluation of the leaves of the five species studied in this work, and these compounds were identified by Balyan and Sarkar [73] and Balyan et al. [74] in jambolan leaves and by Faleiro et al. [41], Saber et al. [42], Santos et al. [50], and Beltrame et al. [44] when studying araçá leaves. Anthocyanins, flavones and flavonones were also identified in leaves of the same plant in the study of Zandoná et al. [43].

Quercetin is an aglycone, which can be found in the glycosylated form bound to different sugars; some examples are isoquercitrin (quercetin-3-*O*-glucoside), quercitrin (quercetin-3-*O*--L-rhamnoside), and rutin (quercetin-3-*O*-rutinoside). All of these compounds are found in extracts of cambuí and gabiroba leaves [51,55].

Fidelis et al. [72], Khan et al. [89], Mahindrakar and Rathod [90], and Andrade et al. [91] when observing the presence of anthocyanins, flavanones, and other compounds, such as epicatechin, ramnetin, and myricetin, reinforce the bioactive potential of jambolan and jabuticaba seeds, which are usually neglected.

Another usually discarded and little used part of the fruit is the peel; however, several studies were selected that aimed to characterize this constituent fraction as a source of flavonoids (Table 1). These compounds are often responsible for the color of the fruit in its different stages of maturation. Regarding araçá, the main flavonoids identified in the peels were anthocyanins [39], while for cambuí fruits, in addition to anthocyanins, catechin was also determined in the work of Santos et al. [54].

When it comes to jabuticaba and jambolan peels, the anthocyanins responsible for the red, purple, and/or blue colors were mostly identified as delphinidin, cyanidin, malvidin, pelargonidin, peonidin, and petunidin. Flavonoids, such as kaempferol, catechin, epicatechin, myricetin, quercetin, rutin and others were also found in of these fruits (Table 2).

Plantagoside (flavanone), hesperetin 7-*O*-glucuronide (flavanonol), apigenin (flavone), taxifolin (flavanone), and isorhamnetin 3-*O*-glucoside (flavonol) were other flavonoids identified in the extracts of the studied fruits, both whole, dried, or lyophilized. Pulps, commonly used in product development, were characterized by Garcia et al. [4] when evaluating cambuí accessions, Alves et al. [56] when studying gabiroba fruits, Dantas et al. [70] when analyzing jabuticaba, and Sousa et al. [87] when studying jambolan, identifying anthocyanins, catechins, procyanidins, kaempferol, diosmetin, and quercetin, among other compounds.

### 2.2. Health Effects

Diets rich in industrialized, refined products, high in sugar, fat, and lacking in vegetables are one of the main risk factors for the populations’ health, since they are associated with delicate health conditions, favoring cardiovascular diseases, diabetes, stroke, obesity, and certain types of cancers, among others [92]. As such, scientific works have focused on the study of the bioactive content of fruits and vegetables, to make them available as an option for consumption and, also, to report their positive health effects, mainly associated with the prevention of chronic non-communicable diseases (Table 3).

Insulin resistance (IR) may be a risk factor for the development of cardiovascular disease and steatosis, which is associated with abdominal obesity, type 2 diabetes, and other syndromes. This pathology may contribute to increased oxidative stress and damage to cell membranes and other functional components, such as proteins and lipids [93].

Due to these issues, Cardoso et al. [93] evaluated the effects of araçá extracts on metabolic parameters and markers of hepatic oxidative stress in an animal model of dexamethasone-induced insulin resistance. The authors observed that anthocyanins were present in the evaluated extracts and that they had a preventive potential against hyperglycemia and hypertriglyceridemia caused by IR, with an antioxidant and protective effect on the formation of reactive oxygen species.

In addition to the properties of araçá, Vinholes et al. [94] concluded that extracts from the genotypes of yellow and red araçás are excellent sources of bioactive compounds, especially anthocyanins, which show promising inhibition of α-glucosidase and help to lower blood glucose in patients with type 2 diabetes mellitus. In turn, Pereira et al. [3] quantified the bioactive compounds present in different parts of the araçá fruit, and evaluated their antioxidant activity and lipase inhibition properties; according to the authors, araçá fruit extracts can be beneficial for the treatment of obesity. Saber et al. [42] verified the efficacy of *P. guajava* and *P. cattleianum* leaf extracts and their nano-liposomes in improving paracetamol-induced hepatotoxicity in rats.

Regginato et al. [5] evaluated gabiroba seed extract, in which it was possible to identify the compound 5,7-dimethoxyflavone, one of the main flavones with biological activities, which include anti-diabetes, anti-obesity, and hypolipidemic activity. According to Arcari et al. [56], gabiroba fruits showed antidiabetic and antioxidant effect properties and can potentially be adopted as part of dietary strategies in the management of the early stages of type 2 diabetes and associated complications.

The antigenotoxic potential of jabuticaba peel extracts was investigated in the work by Marques et al. [95] as inhibitors of phospholipases A2 and proteases. These enzymes are present in snake venom and can act on various components of blood clotting. Results showed aqueous and methanolic extracts were able to modulate the enzymatic activity of snake venom, inhibiting phospholipases and proteases (mainly of the thrombin type). This is due to the presence of phenolic compounds, capable of interacting with catalytic sites of enzymes, leading to a decrease or inhibition of their activities.

Hydroalcoholic extracts of jabuticaba peels were also evaluated in the work by Romão et al. [96]. The authors studied the possible cardioprotective effects of the material in rabbits in the doxorubicin-induced heart failure model. It can be verified that the treatment with *P. cauliflora* extracts induced a cardiorenal protective response, preventing hemodynamic, functional, and remodeling changes. Paula et al. [97] investigated the antioxidant and anti-inflammatory potential of leaves and branches of this same plant and highlighted the great biological activity of these plant material, which are often underutilized and little reported in the literature.

Anthocyanin-rich fractions extracted from jambolan were evaluated in work by Chamnansilpa et al. [101]. The results of this study showed interference of these flavonoids in digestion steps and the absorption of lipids, with inhibition of pancreatic lipase and cholesterol esterase. Additionally, it was found that all extracts could bind primary and secondary bile acids and reduce cholesterol solubility in artificial micelles.

Anti-inflammatory and antinociceptive properties were demonstrated by Qamar et al. [103] and Qamar et al. [6] when evaluating extracts from jambolan fruits in in vivo assays using mice. Anti-inflammatory activity is credited due to synergistic effects of anthocyanins, phenolic acids, and other flavonoids, identified and quantified in *S. cumini* fruit extracts employing HPLC.

The same flavonoid compound may be present in different parts of the plant, as is the case of myricetin, identified in the jambolan fruit in work by Soorya et al. [100] and in its leaves in the studies by Baldissera et al. [98] and Gaspar et al. [99]. Myricetin was associated with potential antiplatelet effects, revealing a new therapeutic perspective for the treatment of thrombotic diseases [99]

Baldissera et al. [98] evaluated functional capacity, phytochemical parameters, oxidative stress, and DNA damage using a crude hydroalcoholic extract of jambolan leaves in diabetic rats. The authors observed that, due to the presence of myricetin glucosides, the extract showed potential hypolipidemic, hypoglycemic, and protective activities against oxidative stress and DNA damage. Still evaluating jambolan leaves, Franco et al. [102] and Borba et al. [104] demonstrated that the antioxidant actions of extracts made with this constituent fraction were associated with the prevention of oxidative processes, glycation, and other inflammatory processes.

### 2.3. Product Development and Flavonoid Preservation

Fruit production is one of the most prominent activities in the Brazilian market, especially when considering the development of new products resulting from the processing of these raw materials [105], as Brazil is currently the third largest producer of fruit in the world, with an average annual production of 45 million metric tons [106].

Fruit growing activity can generate a multiplier effect, with the possibility of moving the economy and promoting the development of stagnant places with few viable resources [107]. Fruit processing aims to minimize seasonality issues and the high perishability of these raw materials, seeking to increase widespread consumption in regions of low production and to improve the conservation conditions of these foods [108].

Linked to these conditions and aiming at the production of sensorially accepted foods with added nutritional value, scientific research promotes the development of juices, purees, jams, ice creams and/or other dairy desserts, and fermented, protein, or isotonic drinks, aiming at the addition of natural ingredients and, consequently, the incorporation of bioactive compounds, such as flavonoids [106,108,109,110,111].

Table 4 summarizes the data referring to the selected articles that had an approach focused on the development and characterization of food products made with the respective fruits researched by this integrative review. There were no articles found approaching this theme for the gabiroba fruit.

Preparation of juices was proposed in the work of Rybka et al. [109] when using cambuí, in Geraldi et al. [108] when using jabuticaba, and in Carvalho et al. [120] when using jambolan. Eight anthocyanins were detected in jambolan juice, namely delphinidin-3,5-diglucoside, cyanidin-3,5-diglucoside, petunidin-3,5-diglucoside, peonidin-3,5-diglucoside, malvidin-3,5-diglucoside, delphinidin-3-glucoside, cyanidin-3-glucoside, and malvidin-3-glucoside.

Anthocyanins were also present in cambuí juice, being quantified (311.7 mg) and expressed in mg of malvidin-3-glucoside per 100 mL of product [109]. In jabuticaba juice, in addition to the presence of anthocyanins, other flavonoids were identified, such as quercetin derivatives, rutin, quercimerithrin, and kaempferol [108].

Ice cream and dairy desserts are products much appreciated by the population, mainly due to their sensory and nutritional characteristics, since they appeal to a diverse audience and because of the presence in their formula of several nutrients, such as proteins, carbohydrates, lipids, calcium, phosphorus, and other minerals [110,123].

Considering this fact, Böger et al. [110] quantified the content of anthocyanins in ice cream, resulting in 10.75 mg of cyanidin-3-glucoside in 100 g of product added with 15% of jabuticaba peel extract. Lino et al. [122], when evaluating the effect of thermosonication on the concentrations of manomeric anthocyanins in dairy desserts developed with jambolan, observed that the process had no significant effect on the content of these constituents.

According to Neves et al. [117], alcoholic beverages comprise the most popular and accepted processed products by the population. Knowledge of the chemical profile, antioxidant capacity, and levels of amino acids and organic acids contribute to intensifying the popularity of these beverages and, therefore, the flavonoids which compose them. Anthocyanins were the group of flavonoids identified in wines [115,118,119], liqueurs [117] and other alcoholic beverages [109], mainly by their glycosidic derivatives. Anthocyanins were also quantified in protein drinks (average of 1.6 mg/100 g) and in isotonic drinks (average of 2.61 mg of cyanidin-3-glucoside in 100 mL) [113,114].

Frozen fruit purees are products widely used in the preparation of other foods, due to their nutritional and functional characteristics, since they are rich in phenolic compounds, especially flavonoids [106]. Stafussa et al. [106] evaluated the phenolic content and biological properties of 10 commercial frozen fruit purees, including araçá and jabuticaba. Flavonoids, such as cyanidin, kaempferol, and quercetin rhamnoside and quercetin were found in jabuticaba purees, while catechin was found in araçá products.

Tea, in general, is one of the most consumed beverages in the world, being a rich source of flavonoids, mainly due to the use of different parts of the plant in the product development, whether including leaves, stems, rhizomes or fruits [124]. Sari et al. [121] proposed the elaboration of teas, produced from jambolan peel, and evaluated the antioxidant and sensory properties of this beverage. It was observed that the product prepared at 50 °C showed high concentrations of anthocyanins and good preference for the color attribute, a parameter associated with the presence of these natural pigments.

Cambuí and jabuticaba processing by-products were used by Rybka et al. [109] and Rodrigues et al. [112] for the development of jellies and microcapsules, respectively, with the microcapsules applied in gelatin, evaluating the color stability of the product and its sensory acceptance. The main flavonoids quantified in these materials were anthocyanins, as well as in flaked jabuticaba, obtained by rotating cylinder drying in the work by Nunes et al. [116].

## 3. Methodology

This integrative review consists of a study based on the collection and analysis of scientific works related to the theme “Flavonoids in plants of the Myrtaceae family”, elaborated from the reading of online journals. The study presented the following steps: (1) formulation of the guiding question; (2) definition of search methods; (3) selection of scientific works; (4) analysis and evaluation of the studies included in the review; (5) presentation of the synthesis of the knowledge produced and published.

A guiding question was proposed while conducting this study, namely “What are the main flavonoids found in certain plants of the Myrtaceae family?”. Data collection took place during the months of February and May 2022 in the following databases: Science Direct, Google Scholar, and Scielo. Basic descriptors used in the research process were as follows: Flavonoid AND Myrtaceae, in addition to specific terms for each plant species, as shown in Table 5. For the Scielo database, the terms Flavonoid AND Myrtaceae were not employed due to greatly restricting the number of results. The same issue occurred with the term Myrtaceae for searches in the Science Direct database.

The following inclusion criteria were defined: studies published in the databases, in the period between 2016 to 2022, presented in full text, in English, Portuguese, or Spanish, and whose title and/or abstract referred to the topic of flavonoids in plants of the Myrtaceae family, such as Araçá (*P. cattleianum*), Cambuí (*M. floribunda*), Gabiroba (*C. xanthocarpa*), Jabuticaba (*P. cauliflora*), and Jambolan (*S. cumini*).

Initially, a critical and reflective reading of the titles and abstracts was performed, selecting those that met the defined inclusion criteria. The second stage of the study comprised a complete reading of the selected articles, extracting from them the evidence related to flavonoids in each species studied. In this phase, for better organization of the analysis through the exploratory reading of each article, those that presented elements of interest were identified; however, at this step of the process, some scientific review works were also excluded. The selection of scientific studies over the years in relation to the evaluated fruits were graphically represented by a bubble chart developed in Microsoft PowerPoint (2013).

## 4. Conclusions

It was, therefore, possible to observe the importance of plants in the Myrtaceae family in terms of their phytochemical composition in relation to flavonoids, positive health effects, and the possibilities for their use in product development. A more significant number of scientific works associated with jambolan and jabuticaba were selected, with in vivo and in vitro experiments demonstrating these raw materials’ bioactive potential. This way, an association was possible with specific health benefits, such as antioxidant, cardioprotective, antidiabetic, and anti-inflammatory activities.

The preservation of flavonoids in jams, juices, wines, and other foods can also be observed, with anthocyanins being the predominant chemical class. Additionally, the integrative review employment as a methodology for this study proved relevant for achieving the objective. It guides the research practice and encompasses several scientific works on a subject.

## Figures and Tables

**Table 1:** Synthesis of selected works with the approach of phytochemical characterization of the different constituent fractions of plants and their flavonoids.

Vegetable Fraction	Flavonoids	References
**Araçá**		
**Leaf**	Catechin, gallocatechin, epigallocatechin, hesperetin-7-*O*-glucuronide, plantagoside, quercetin, reynoutrin, luteolin-7-glucuronide, quercitrin, myricetin, apigenin-7-*O*-glucoside, peonidin-3-glucoside, hispidulin, gardenin A, 8-hydroxy-5-methoxyflavanone, and 3′,4′-dimethoxy-7-hydroxyflavone	[41,42,43,44]
**Fruit**	Taxifolin, gallocatechin, catechin, epicatechin derivatives, myricetin, naringenin, quercetin, anthocyanins, delphinidin, cyanidin, cyanidin-3-glucoside, cyanidin-3,5-diglucoside, malvidin-3-glucoside, pinobanksin, isoquercitrin, isorhamnetin, luteolin, and kaempferol	[3,45,46,47,48,49]
**Peel**	Anthocyanins	[39]
**Cambuí**		
**Leaf**	Rutin, epigallocatechin, catechin, isoquercitrin, quercitrin, myricetin derivatives, procyanidin B dimer, and kaempferol-*O*-rhamnoside	[50,51]
**Fruit**	Rutin and anthocyanins	[52,53]
**Peel**	Catechin and anthocyanins	[54]
**Pulp**	Reynoutrin, quercetin, quercitrin, myricetin derivatives, myricthrin, procyanidin A2, cyanidin-3-*O*-rutinoside, methyldihydromyricetin, catechin, diosmetin, petunidin, epicatechin gallate, delphinidin hexoside, apigenin 7-O-neohesperidoside, and rutin	[4]
**Gabiroba**		
**Leaf**	Quercetin, luteolin, vitexin, isoquercetin, and quercitrin	[55]
**Fruit**	Catechin, epicatechin, quercetin, isorhamnetin 3-*O*-glucoside, naringenin, kaempferol, and apigenin	[56]
**Pulp**	Catechin	[57]
**Jabuticaba**		
**Leaf**	Quercetin, myricitrin, catechin, luteolin 7-glucuronide, eriocitrin, and hesperetin	[7]
**Fruit**	Delphinidin, cyanidin, pelargonidin, peonidin, myricetin derivatives, quercetin derivatives, and catechin	[58]
**Peel**	Quercetin, quercetin derivatives, catechin, myricetin, myricetin derivatives, epicatechin, gallocatechin, epicatechin gallate, anthocyanins, delphinidin, delphinidin-3-glucoside, cyanidin, cyanidin-3-glucoside, malvidin, pelargonidin, pelargonidin-3-glucoside, peonidin, peonidin-3-glucoside, and petunidin	[37,59,60,61,62,63,64,65,66,67,68,69]
**Pulp**	Catechin, procyanidin B1, procyanidin B2, anthocyanins, cyanidin-3-glucoside, and kaempferol	[70,71]
**Seed**	Quercetin, rutin, procyanidin A2, malvidin-3,5-diglucoside, and cyanidin-3-glucoside	[72]
**Jambolan**		
**Leaf**	Catechin, myricetin derivatives, quercetin, and epicatechin	[73,74]
**Plant**	Rutin, catechin, myricetin, and quercetin	[75]
**Fruit**	Catechin, epigallocatechin gallate, isoquercitrin, isorhamnetin, kaempferol, myricetin, luteolin, naringenin, quercetin, anthocyanins, cyanidin, cyanidin-3-glucoside, malvidin 3-glucoside, delphinidin, epicatechin, rutin, and pinobanksin	[32,76,77]
**Peel**	Anthocyanins, delphinidin-3,5-diglucoside, cyanidin-3,5-diglucoside, petunidin-3,5-diglucoside, peonidin-3,5-diglucoside, and malvidin-3,5-diglucoside	[78,79,80,81,82]
**Pulp**	Quercetin, catechin, rutin, myricetin, anthocyanins, delphinidin, delphinidin-3,5-diglucoside, cyanidin, cyanidin-3,5-diglucoside, cyanidin-3-glucoside, petunidin, petunidin-3,5-diglucoside, petunidin-3-glucoside, peonidin, peonidin-3,5-diglucoside, peonidin-3-glucoside, malvidin, malvidin-3,5-diglucoside, malvidin-3-glucoside, and epigallocatechin	[83,84,85,86,87,88]
**Seed**	Catechin, naringin, rutin, myricetin, epicatechin gallate, ramnetin, and epigallocatechin gallate	[89,90,91]

(Authors 2022).

**Table 2:** Basic structures of some flavonoid classes verified in selected studies.

Flavanone	
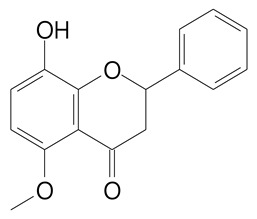	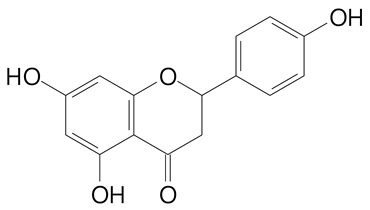
8-Hydroxy-5-methoxyflavanone (270.3 g/mol)	Naringenin (272.3 g/mol)
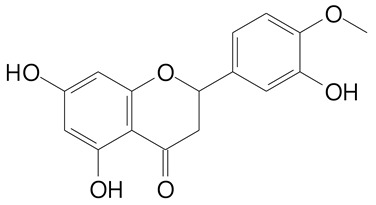	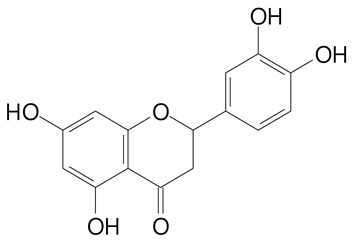
Hesperetin (302.3 g/mol)	Taxifolin (304.2 g/mol)
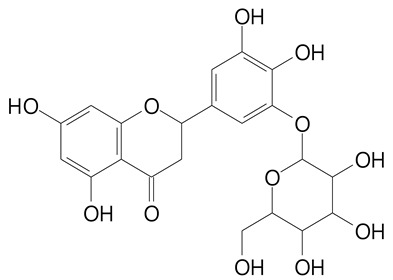	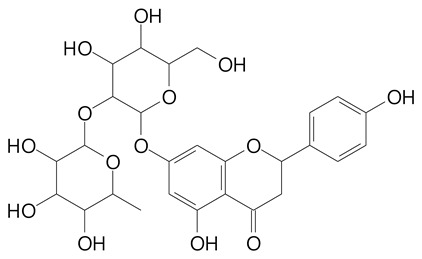
Plantagoside (466.4 g/mol)	Naringin (580.5 g/mol)
**Flavanol**	
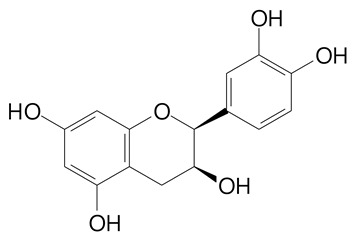	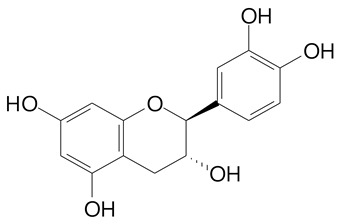
Epicatechin (290.3 g/mol)	Catechin (290.3 g/mol)
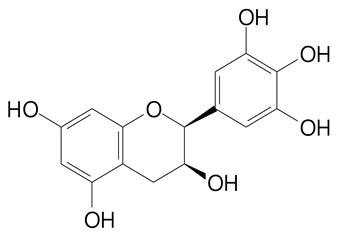	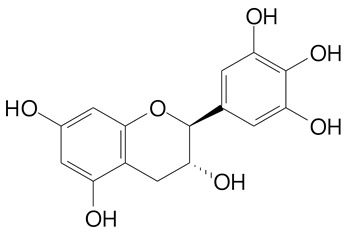
Epigallocatechin (306.3 g/mol)	Gallocatechin (306.3 g/mol)
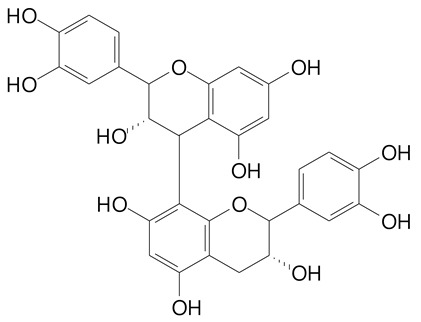	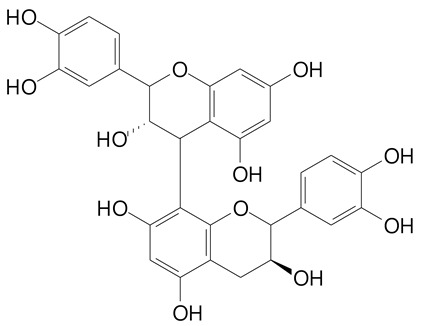
Procyanidin B1 (578.5 g/mol)	Procyanidin B2 (578.5 g/mol)
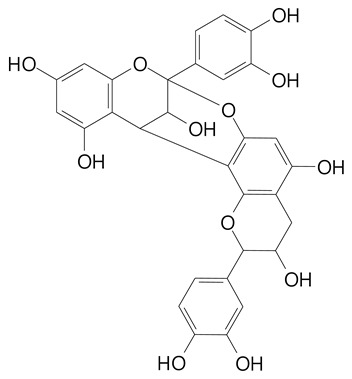	
Procyanidin A2 (576.5 g/mol)	
**Flavanonol**	
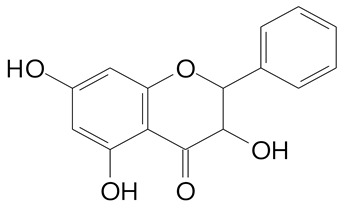	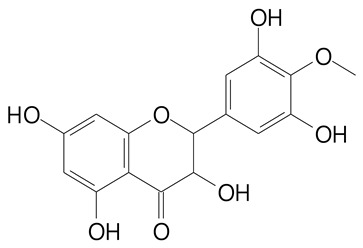
Pinobanksin (272.2 g/mol)	Methyldihydromyricetin (334.3 g/mol)
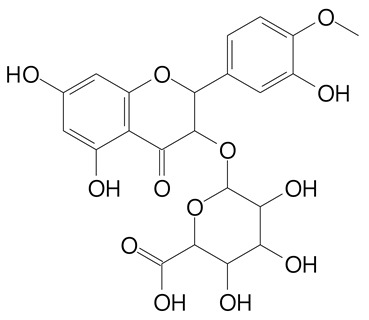	
Hesperetin-7-*O*-glucuronide (466.4 g/mol)	
**Flavonol**	
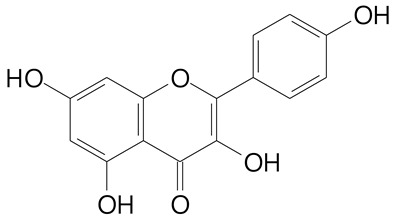	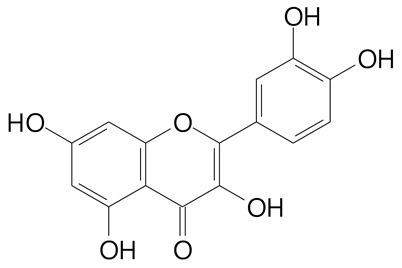
Kaempferol (286.2 g/mol)	Quercetin (302.2 g/mol)
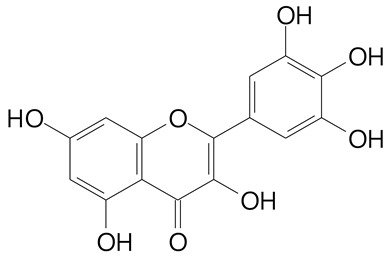	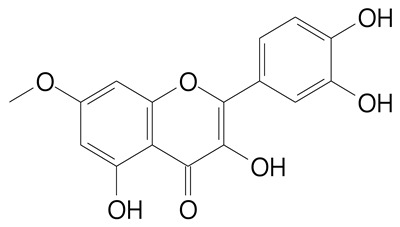
Myricetin (318.2 g/mol)	Rhamnetin (316.3 g/mol)
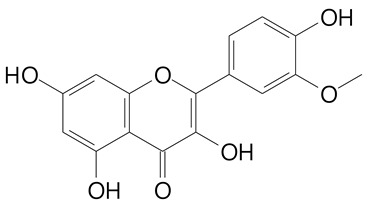	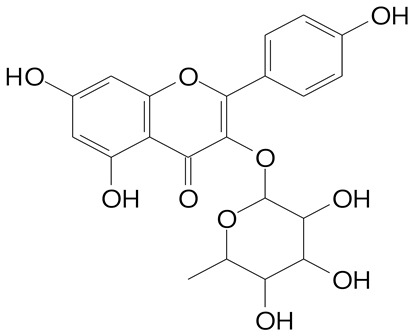
Isorhamnetin (316.3 g/mol)	Kaempferol-*O*-rhamnoside (432.4 g/mol)
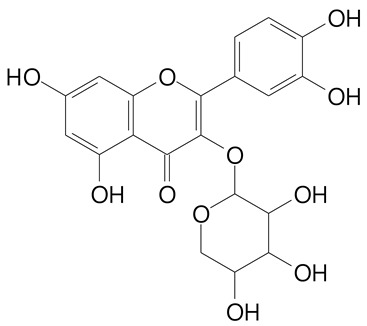	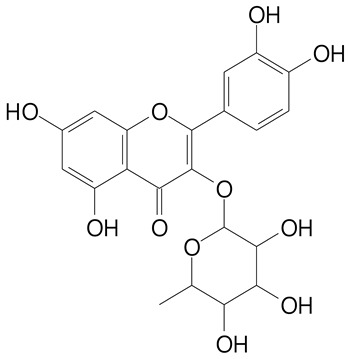
Reynoutrin (434.3 g/mol)	Quercitrin (448.4 g/mol)
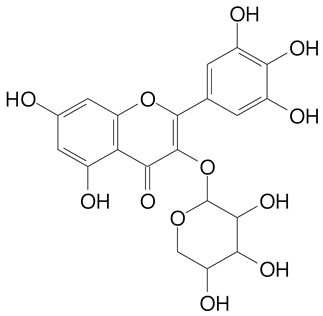	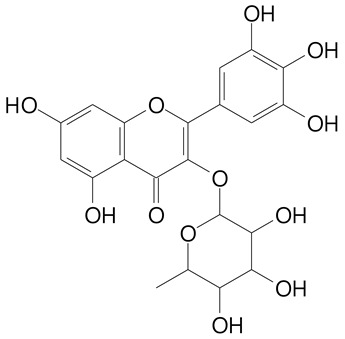
Myricetin 3-arabinoside (450.3 g/mol)	Myricitrin (464.4 g/mol)
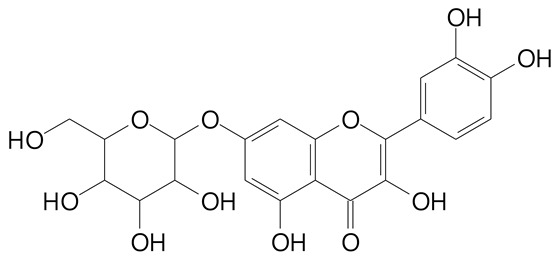	
Quercimeritrin (464.4 g/mol)	
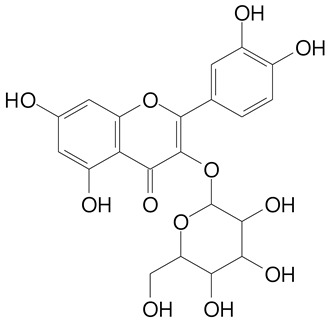	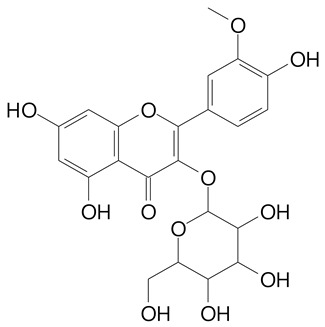
Isoquercitrin (464.4 g/mol)	Isorhamnetin-3-*O*-glucoside (478.4 g/mol)
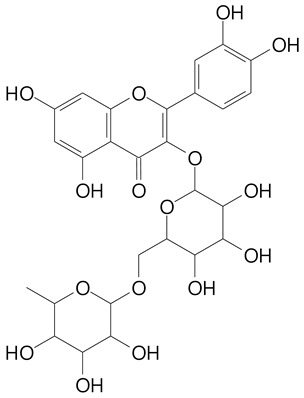	
Rutin (610.5 g/mol)	
**Flavone**	
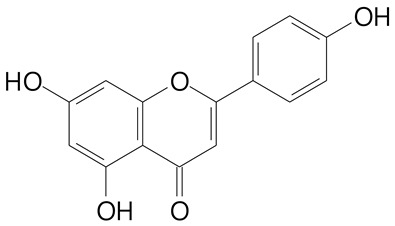	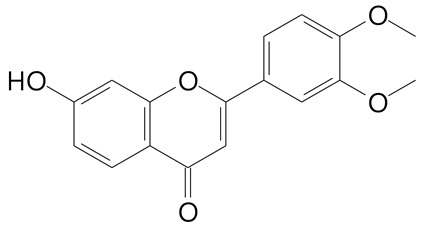
Apigenin (270.2 g/mol)	3′,4′-dimethoxy-7-hydroxyflavone (298.2 g/mol)
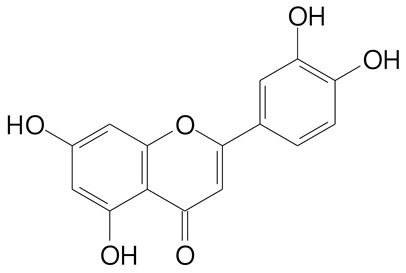	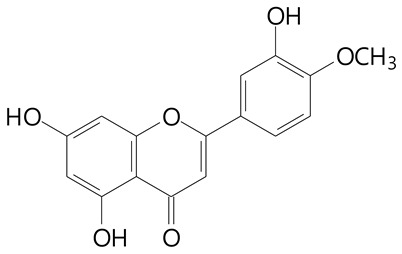
Luteolin (286.2 g/mol)	Diosmetin (300.3 g/mol)
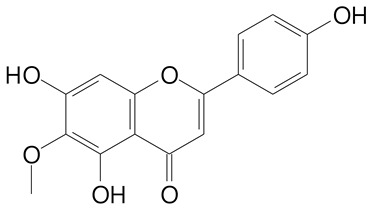	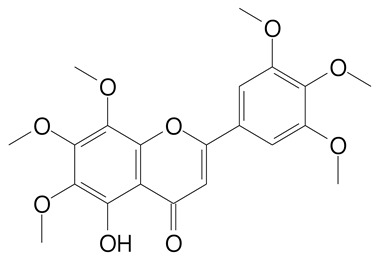
Hispidulin (300.3 g/mol)	Gardenin A (418.4 g/mol)
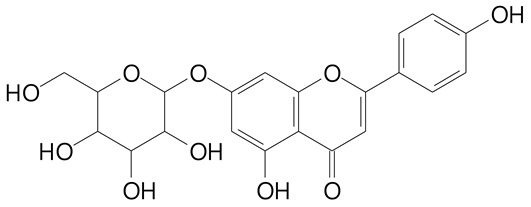 Apigenin 7-O-glucoside (432.4 g/mol)	
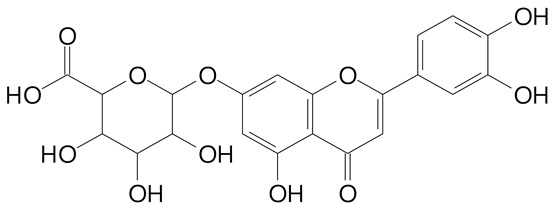 Luteolin 7-glucuronide (462.4 g/mol)	
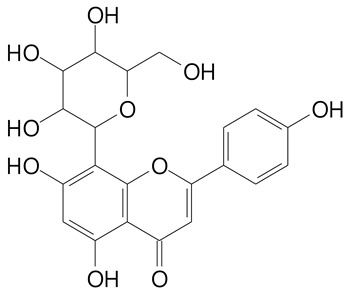	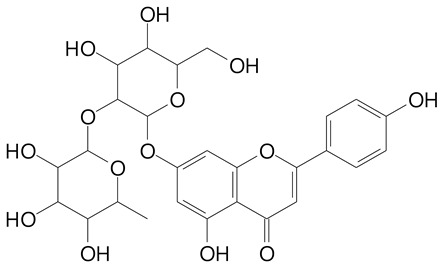
Vitexin (432.4 g/mol)	Apigenin 7-*O*-neohesperidoside (578.5 g/mol)
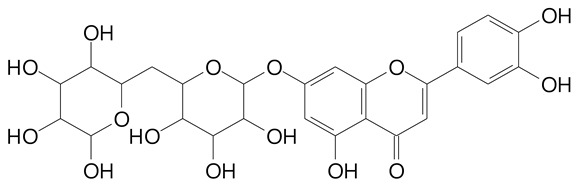	
Eriocitrin (596.5 g/mol)	
**Anthocyanins**	
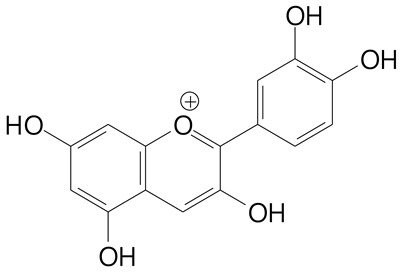	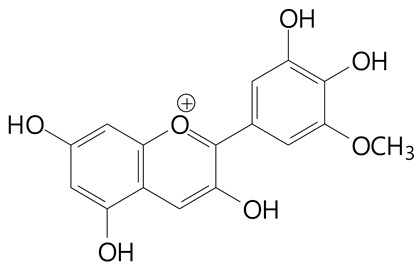
Cyanidin (287.2 g/mol)	Petunidin (317.3 g/mol)
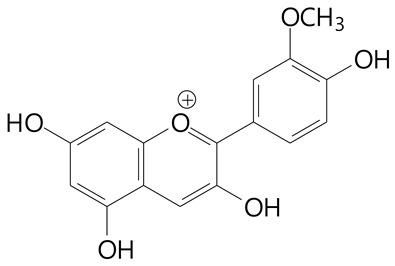	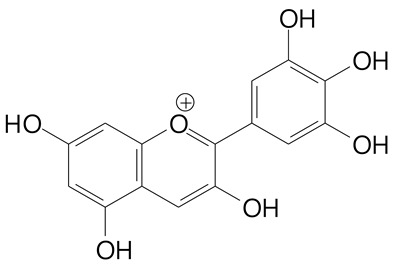
Peonidin (301.3 g/mol)	Delphinidin (338.7 g/mol)
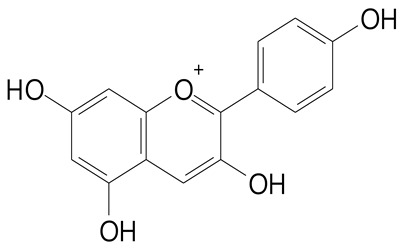	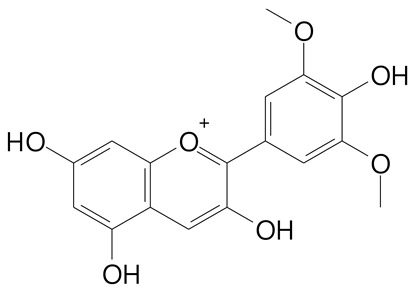
Pelargonidin (271.2 g/mol)	Malvidin (331.3 g/mol)
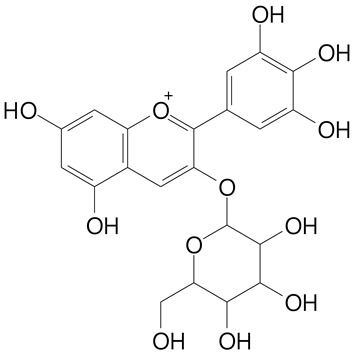	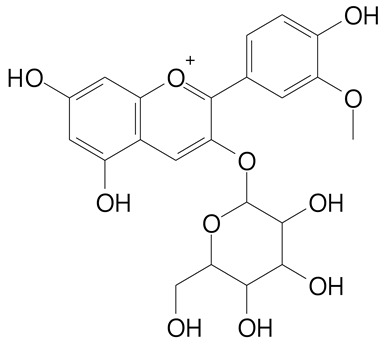
Delphinidin 3-glucoside (465.4 g/mol)	Peonidin-3-glucoside (463.4 g/mol)
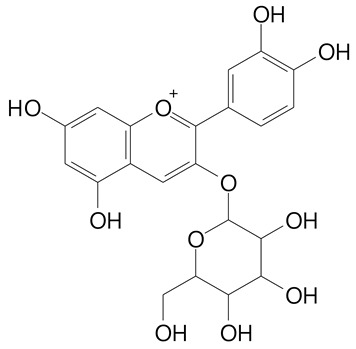	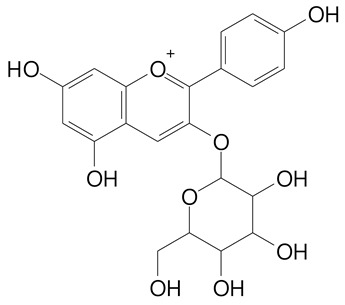
Cyanidin 3-glucoside (484.8 g/mol)	Pelargonidin 3-glucoside (433.4 g/mol)
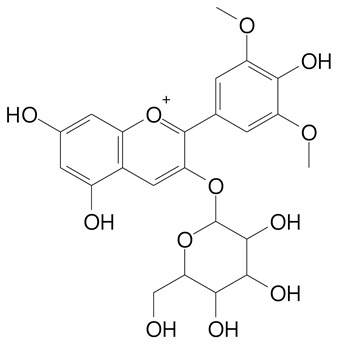	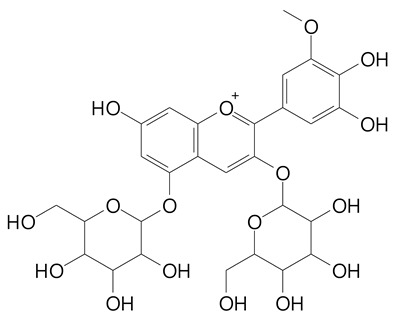
Malvidin 3-glucoside (528.9 g/mol)	Petunidin 3,5-diglucoside (677.0 g/mol)
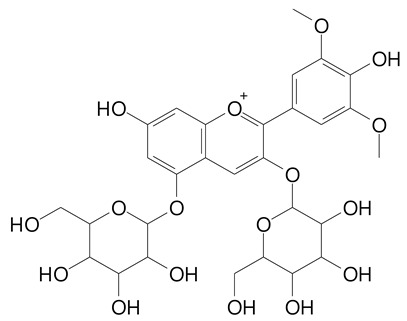	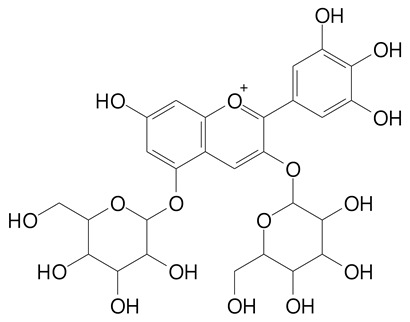
Malvidin-3,5-*O*- diglucoside (655.6 g/mol)	Delphinidin 3,5-diglucoside (627.5 g/mol)
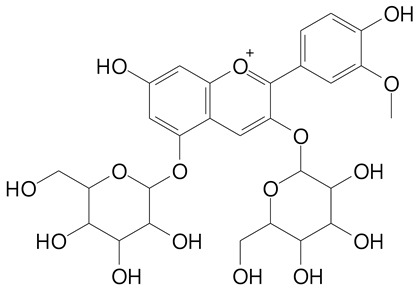	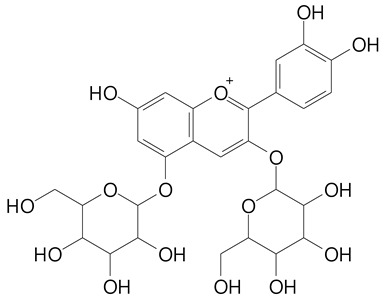
Peonidin 3,5-diglucoside (625.6 g/mol)	Cyanidin 3,5-diglucoside (611.5 g/mol)
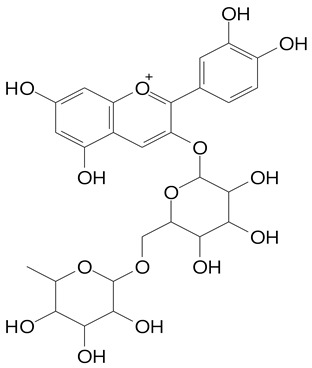	
Cyanidin 3-O-rutinoside (595.5 g/mol)	

(Authors 2022).

**Table 3:** Synthesis of selected works with the main flavonoids identified in extracts and their positive health effects.

References	Experiment	Health Effects
**Araçá**		
[3]	Lyophilized araçá samples (250 mg) were stirred in 10 mL of ethanol (50 %) (1:40, *w/v*). The extracted araçá samples (2.5 mg/mL) were added separately to the lipase mixture. Absorbance was recorded in a microplate reader and compared with that of the lipase mixture without any extract (control).	Beneficial for the treatment of obesity
[42]	Adult male albino Wistar rats weighing 120–140 g body weight (b.wt) were divided into experimental groups, as follows: (1) control group—received 0.5% carboxy methyl cellulose, orally; (2) paracetamol group—rats were fasted for 18 h and paracetamol was orally administered in a single dose of (600 mg/kg); (3) silymarin (50 mg/kg b.wt) and paracetamol; (4–9) extract or formula and paracetamol were pre-treated with the chloroform–methanol (80:20) extract of *P. guajava* L. (PG) and *P. cattleianum* (PC) (250 and 500 mg/kg b.wt) and their formulae PG and PC (500 mg/kg b.wt), two weeks before induction of hepatic damage.	Antioxidant and hepatoprotective activities
[93]	Forty-eight adults male Wistar rats were obtained and divided into experimental groups, as follows: (1) control/vehicle; (2) control/*P. cattleianum*; (3) dexamethasone/vehicle; (4) dexamethasone/*P. cattleianum*. Groups 1 and 3 received distilled water and groups 2 and 4 received 200 mg/kg/day *P. cattleianum.*	Antioxidant, antihyperglycemic, and antidyslipidemic effects
[94]	For the evaluation of the anti-hyperglycaemic and antioxidant potential of fruit extracts, in vitro assays were performed by applying spectrophotometrics. The IC50 values were calculated using at least five concentrations for each extract.	Antihyperglycemic, antioxidant properties, and α-glucosidase inhibitors
**Gabiroba**		
[5]	Rats were divided into groups of six animals, as follows: (1) bormal rats that received 1% tween 80 solution in saline (0.5 mL/100 g bw); (2) hyperglycemic rats that received glucose solution (4 g/kg; 8.9 M); (3)–(5) hyperglycemic rats that received glucose solution plus *C. xanthocarpa* seeds extract solution (200, 400, or 800 mg/kg, respectively); (6) Hyperglycemic rats treated with glibenclamide (10 mg/kg) and glucose solution, by gavage.	Antidiabetic and hypolipidemic potential
**Jabuticaba**		
[37]	Cell lineages of prostate (DU-145) and breast cancers (MDA-MB-231) were plated at a concentration of 9 × 104 cells per well (well trays = 96) and filled with 100 μL of culture medium (with fetal bovine serum—FBS). The culture medium was changed, and the cells were subjected to treatments with jabuticaba peel extracts at concentrations of 2.5, 25, 50, and 250 μg.mL^−1^. Cells were treated with doxorubicin and the extracts were diluted in culture medium without FBS, and the final concentration of water/dimethylsulfoxide (DMSO) was at most 0.2% to avoid harming cellular viability. Control groups consisted of cells cultivated in culture medium with DMSO and without FBS.	Antiproliferative activity in tumor cell lines
[95]	The aqueous and methanolic extracts of jabuticaba skin flour were previously incubated with the venoms of *Bothrops moojeni* and *Lachesis muta muta* at the proportions of 1:0.5; 1:1; 1:2.5, and 1:5 (venom:extract, *w:w*). Tubes containing citrated plasma (200 μL) were kept in a 37 °C bath. Incubated samples were added to the plasma and time was recorded until the formation of the clot. Controls containing only the extracts were also carried out.	Potential antigenotoxic and modulator of processes related to hemostasis
[96]	Twenty-week-old female New Zealand rabbits (weighing 2.5–3.0 kg) were randomly assigned to five experimental groups (*n* = 6/group), as follows: (1) naive (the rabbits received a placebo [distilled water] and were treated with vehicle [filtered water]); (2) negative control (the rabbits received doxorubicin and were treated with vehicle [filtered water]); (3) EEPC 75 (the rabbits received doxorubicin and were treated with 75 mg/kg EEPC); (4) EEPC 150 (the rabbits received doxorubicin and were treated with 150 mg/kg EEPC); (5) ENAL 5 (the rabbits received doxorubicin and were treated with 5 mg/kg enalapril).	Cardioprotective effects
[97]	Stock solutions at 1 mg/mL of ethanolic extracts of leaves (EEL) and branches (EEB) were prepared in MeOH and then diluted to concentrations between 1000 and 31.25 μg/mL. The measurements were obtained at 0–15 min intervals during 2 h of reaction, and the plate was incubated at 45 °C. The same was carried out for positive controls, rutin and quercetin, and the negative control (vehicle).	Anti-inflammatory and antioxidant properties
**Jambolan**		
[98]	Male Wistar rats were used, at 60 days of age, and weighing 200–270 g. They were divided into five groups, as follows: (1) normoglycemic controls (CONT, *n* = 8); (2) diabetic controls (D-CONT, *n* = 8); (3) diabetics treated with a crude hydroalcoholic extract of *S. cumini* leaves (D+EBH, *n* = 8); (4) trained diabetics (D+TAC, *n* = 8) and (5) diabetics treated with the extract and trained (D+EBH+TAC, *n* = 5).	Protection against DNA damage
[99]	Healthy female Swiss mice (*Mus musculus*) 10–12 weeks of age and weighing 30–35 g were given myricetin at 25 mg/kg or 50 mg/kg or vehicle control for three consecutive days through oral gavage.	Platelet thiol isomerase inhibitors (PDI and ERp5) activities
[100]	Physicochemical, ADMET (absorption, distribution, metabolism, excretion, and toxicity), and druggability properties of myricetin—a key flavonoid compound in *S. cumini*—have been evaluated.	Regulation of metabolic inflammation
[101]	The porcine pancreatic lipase (7.5 mg/mL) and 0.2 mM 4-MUO were prepared in 0.1 M PBS. To determine the lipase activity, the solution of anthocyanin-rich extract (5 μL) was mixed with 50 μL of 4-MUO solution. Then, the enzyme solution (45 μL) was added to the mixture to initiate the reaction. The mixture was immediately incubated before adding 100 μL of 0.1 M sodium citrate to stop the reaction. The absorbance of fluorescence was read at the excitation wavelength of 355 nm and 460 nm. Orlistat in 1% DMSO was used as a positive control.	Interference with the absorption of lipids and cholesterol
[102]	Ethanolic extract of *S. cumini* leaves (EE-SCL)/quercetin (also used as a positive control) were diluted in ethanol at concentration of 30 mg mL-1 for IC50 assessment and phosphate buffer was used as negative control.	Potential against oxidation, glycation, inflammation, and digestive enzyme catalysis
[103]	The in vitro anti-inflammatory activities of *S. cumini* fruit extracts were evaluated using membrane stabilization, egg albumin denaturation, and bovine serum albumin denaturation assays. The in vivo anti-inflammatory activity was also assessed, using murine models of carrageenan, formaldehyde, and PGE2 induced paw edema.	Anti-inflammatory properties in vivo and in vitro
[104]	Ethanolic extract of *S. cumini* at initial concentrations of 125, 250, 500, 750, and 1000 mg/mL, rivastigmine (100 mg/mL, positive control) or water (control) and 10 mL of acetylcholinesterase (1 U/mL) were pipetted in triplicate, in microplates containing 5,5-dithiobis-2-nitrobenzoic acid (DTNB, 0.33 mM) in sodium phosphate buffer and incubated. After that, 10 mL of acetylthiocholine iodide was added to each sample with a multichannel pipette and the absorbance was monitored at 412 nm for 20 min in a spectrophotometer.	In vitro inhibition of acetylcholinesterase and monoamine oxidase
[6]	Forty Swiss albino mice of both genders were divided into eight groups (five per group), as follows: a control group that received normal saline), indomethacin group (100 mg/kg), dichloromethane, methanol, and 50% methanol (treated with 100 and 200 mg/kg extract’s doses). After intraperitoneal administration of the test sample, 250 μL of 2.5% formalin solution was injected into plantar aponeurosis surface of the right hind paw of each mice and the licking responses of the animals were observed at early neurogenic pain phase after 0–5 min and the later anti-inflammatory pain stage after 20–25 min.	Antinociceptive effect and anti-inflammatory potential

(Authors 2022).

**Table 4:** Synthesis of selected works with the main flavonoids identified in extracts and their positive health effects.

Fruit	Products	Flavonoids	References
**Araçá**	Purees	Catechin	[106]
**Cambuí**	Juice	Anthocyanins	[109]
	Jams		
	Fermented drink		
**Jabuticaba**	Microcapsule	Anthocyanins	[112]
	Ice cream	Anthocyanins	[110]
	Protein drink	Anthocyanins	[113]
	Isotonic drink	Anthocyanins	[114]
	Purees	Cyanidin hexoside, kaempferol hexoside, and quercetin derivatives	[106]
	Juice	Cyanidin-3-glucoside, quercetin derivatives, rutin, kaempferol, and quercimerithrin	[108]
	Wine	Anthocyanins	[115]
	Flakes	Anthocyanins	[116]
	Fermented drink	Anthocyanins	[111]
	Liqueur	Peonidin-3-glucoside and cyanidin-3-glucoside	[117]
**Jambolan**	Wine	Anthocyanins, delphidin-3-glucoside, petunidin-3,5-diglucoside, delphinidin-3,5-diglucoside, peonidin 3,5-diglucoside, and cyanidin-3,5-diglucoside	[118,119]
	Juice	Delphinidin-3,5-diglucoside, cyanidin-3,5-diglucoside, petunidin-3,5-diglucoside, peonidin-3,5-diglucoside, malvidin-3,5-diglucoside, delphinidin-3-glucoside, cyanidin-3- glucoside, and malvidin-3-glucoside	[120]
	Tea	Anthocyanins	[121]
	Dairy dessert	Anthocyanins	[122]

(Authors 2022).

**Table 5:** Synthesis of selected works with the main flavonoids identified in extracts and their positive health effects.

Database	Search Strategy	Number of Works
**Google Scholar**	Class of flavonoid AND Myrtaceae family AND Jambolão OR *Syzygium cumini* Class of flavonoid AND Myrtaceae family AND Jabuticaba OR *Plinia cauliflora* Class of flavonoid AND Myrtaceae family AND Cambuí OR *Myrciaria floribunda* Class of flavonoid AND Myrtaceae family AND Araçá OR *Psidium cattleianum* Class of flavonoid AND Myrtaceae family AND Gabiroba OR *Campomanesia xanthocarpa*	**1751**
***Scielo***	(Jambolão) OR (*Syzygium cumini*) (Jabuticaba) OR (*Plinia cauliflora*) (Cambuí) OR (*Myrciaria floribunda*) (Araçá) OR (*Psidium cattleianum*) (Gabiroba) OR (*Campomanesia xanthocarpa*)	**121**
***Science*** ***Direct***	Jambolão OR *Syzygium cumini* AND flavonoids Jabuticaba OR *Plinia cauliflora* AND flavonoids Cambuí OR *Myrciaria floribunda* AND flavonoids Araçá OR *Psidium cattleianum* AND flavonoids Gabiroba OR *Campomanesia xanthocarpa* AND flavonoids	**644**

(Authors 2022).

## Data Availability

All data are contained within the article.
